# Incorporating biological prior knowledge for Bayesian learning via maximal knowledge-driven information priors

**DOI:** 10.1186/s12859-017-1893-4

**Published:** 2017-12-28

**Authors:** Shahin Boluki, Mohammad Shahrokh Esfahani, Xiaoning Qian, Edward R Dougherty

**Affiliations:** 1 Department of Electrical and Computer Engineering, Texas A&M University, MS3128 TAMU, College Station, 77843 TX USA; 2 Division of Oncology and Center for Cancer Systems Biology, Stanford School of Medicine, 291 Campus Drive, Stanford, 94305 CA USA

**Keywords:** Optimal Bayesian classification, Prior construction, Biological pathways, Probabilistic Boolean networks

## Abstract

**Background:**

Phenotypic classification is problematic because small samples are ubiquitous; and, for these, use of prior knowledge is critical. If knowledge concerning the feature-label distribution – for instance, genetic pathways – is available, then it can be used in learning. Optimal Bayesian classification provides optimal classification under model uncertainty. It differs from classical Bayesian methods in which a classification model is assumed and prior distributions are placed on model parameters. With optimal Bayesian classification, uncertainty is treated directly on the feature-label distribution, which assures full utilization of prior knowledge and is guaranteed to outperform classical methods.

**Results:**

The salient problem confronting optimal Bayesian classification is prior construction. In this paper, we propose a new prior construction methodology based on a general framework of constraints in the form of conditional probability statements. We call this prior the *maximal knowledge-driven information prior* (MKDIP). The new constraint framework is more flexible than our previous methods as it naturally handles the potential inconsistency in archived regulatory relationships and conditioning can be augmented by other knowledge, such as population statistics. We also extend the application of prior construction to a multinomial mixture model when labels are unknown, which often occurs in practice. The performance of the proposed methods is examined on two important pathway families, the mammalian cell-cycle and a set of p53-related pathways, and also on a publicly available gene expression dataset of non-small cell lung cancer when combined with the existing prior knowledge on relevant signaling pathways.

**Conclusion:**

The new proposed general prior construction framework extends the prior construction methodology to a more flexible framework that results in better inference when proper prior knowledge exists. Moreover, the extension of optimal Bayesian classification to multinomial mixtures where data sets are both small and unlabeled, enables superior classifier design using small, unstructured data sets. We have demonstrated the effectiveness of our approach using pathway information and available knowledge of gene regulating functions; however, the underlying theory can be applied to a wide variety of knowledge types, and other applications when there are small samples.

## Background

Small samples are commonplace in phenotypic classification and, for these, prior knowledge is critical [1, 2]. If knowledge concerning the feature-label distribution is available, say, genetic pathways, then it can be used to design an optimal Bayesian classifier (OBC) for which uncertainty is treated directly on the feature-label distribution. As typical with Bayesian methods, the salient obstacle confronting OBC is prior construction. In this paper, we propose a new prior construction framework to incorporate gene regulatory knowledge via general types of constraints in the form of probability statements quantifying the probabilities of gene up- and down-regulation conditioned on the regulatory status of other genes. We extend the application of prior construction to a multinomial mixture model when labels are unknown, a key issue confronting the use of data arising from unplanned experiments in practice.

Regarding prior construction, E. T. Jaynes has remarked [3], “…there must exist a general formal theory of determination of priors by logical analysis of prior information – and that to develop it is today the top priority research problem of Bayesian theory”. It is precisely this kind of formal structure that is presented in this paper. The formal structure involves a constrained optimization in which the constraints incorporate existing scientific knowledge augmented by slackness variables. The constraints tighten the prior distribution in accordance with prior knowledge, while at the same time avoiding inadvertent over restriction of the prior, an important consideration with small samples.

Subsequent to the introduction of Jeffreys’ non-informative prior [4], there was a series of information-theoretic and statistical methods: Maximal data information priors (MDIP) [5], non-informative priors for integers [6], entropic priors [7], reference (non-informative) priors obtained through maximization of the missing information [8], and least-informative priors [9] (see also [10–12] and the references therein). The principle of maximum entropy can be seen as a method of constructing least-informative priors [13, 14], though it was first introduced in statistical mechanics for assigning probabilities. Except in the Jeffreys’ prior, almost all the methods are based on optimization: max- or min-imizing an objective function, usually an information theoretic one. The least-informative prior in [9] is found among a restricted set of distributions, where the feasible region is a set of convex combinations of certain types of distributions. In [15], several non-informative and informative priors for different problems are found. All of these methods emphasize the separation of prior knowledge and observed sample data.

Although the methods above are appropriate tools for generating prior probabilities, they are quite general methodologies without targeting any specific type of prior information. In that regard, the problem of prior selection, in any Bayesian paradigm, is usually treated conventionally (even “subjectively”) and independent of the real available prior knowledge and sample data.

**Fig. 1:**
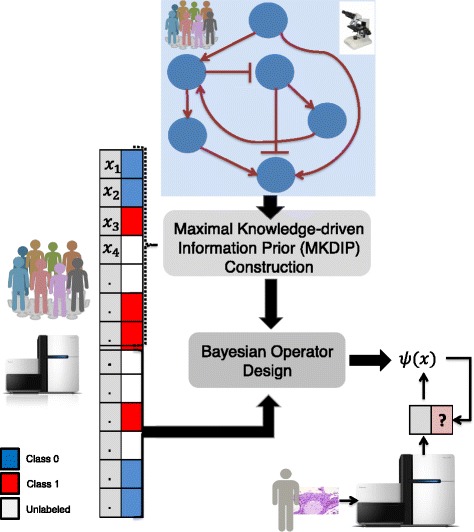
A schematic illustration of the proposed Bayesian prior construction approach for a binary-classification problem. Information contained in the biological signaling pathways and their corresponding regulating functions is transformed to prior probabilities by MKDIP. Previously observed sample points (labeled or unlabeled) are used along with the constructed priors to design a Bayesian classifier to classify a new sample point (patient)

Figure 1 shows a schematic view of the proposed mechanism for Bayesian operator design.

The *a priori* knowledge in the form of graphical models (e.g., Markov random fields) has been widely utilized in covariance matrix estimation in Gaussian graphical models. In these studies, using a given graphical model illustrating the interactions between variables, different problems have been addressed: e.g., constraints on the matrix structure [16, 17] or known independencies between variables [18, 19]. Nonetheless, these studies rely on a fundamental assumption: the given prior knowledge is complete and hence provides one single solution. However, in many applications including genomics, the given prior knowledge is uncertain, incomplete, and may be inconsistent. Therefore, instead of interpreting the prior knowledge as a single solution, e.g., a single deterministic covariance matrix, we aim at constructing a prior distribution on an uncertainty class.

In a different approach to prior knowledge, gene-gene relationships (pathway-based or protein-protein interaction (PPI) networks) are used to improve classification accuracy [20–26], consistency of biomarker discovery [27, 28], accuracy of identifying differentially expressed genes and regulatory target genes of a transcription factor [29–31], and targeted therapeutic strategies [32, 33]. The majority of these studies utilize gene expressions corresponding to sub-networks in PPI networks, for instance: mean or median of gene expression values in gene ontology network modules [20], probabilistic inference of pathway activity [24], and producing candidate sub-networks via a Markov clustering algorithm applied to high quality PPI networks [26, 34]. None of these methods incorporate the regulating mechanisms (activating or suppressing) into classification or feature-selection to the best of our knowledge.

The fundamental difference of the work presented in this paper is that we develop machinery to transform knowledge contained in biological signaling pathways to prior probabilities. We propose a general framework capable of incorporating any source of prior information by extending our previous prior construction methods [35–37]. We call the final prior distribution constructed via this framework, a *maximal knowledge-driven information prior* (MKDIP). The new MKDIP construction constitutes two steps: (1) Pairwise and functional information quantification: information in the biological pathways is quantified by an information theoretic formulation. (2) Objective-based Prior Selection: combining sample data and prior knowledge, we build an objective function, in which the expected mean log-likelihood is regularized by the quantified information in step 1. As a special case, where we do not have any sample data, or there is only one data point available for constructing the prior probability, the proposed framework is reduced to a regularized extension of the maximum entropy principle (MaxEnt) [38].

Owing to population heterogeneity we often face a *mixture model*, for example, when considering tumor sample heterogeneity where the assignment of a sample to any subtype or stage is not necessarily given. Thus, we derive the MKDIP construction and OBC for a mixture model. In this paper, we assume that data are categorical, e.g. binary or ternary gene-expression representations. Such categorical representations have many potential applications, including those wherein we only have access to a coarse set of measurements, e.g. epifluorescent imaging [39], rather than fine-resolution measurements such as microarray or RNA-Seq data. Finally, we emphasize that, in our framework, no single model is selected; instead, we consider all possible models as the uncertainty class that can be representative of the available prior information and assign probabilities to each model via the constructed prior.

## Methods

### Notation

Boldface lower case letters represent column vectors. Occasionally, concatenation of several vectors is also shown by boldface lower case letters. For a vector ***a***, *a*_0_ represents the summation of all the elements and *a*_*i*_ denotes its *i*-th element. Probability sample spaces are shown by calligraphic uppercase letters. Uppercase letters are for sets and random variables (vectors). Probability measure over the random variable (vector) *X* is denoted by *P*(*X*), whether it be a probability density function or a probability mass function. *E*_*X*_[*f*(*X*)] represents the expectation of *f*(*X*) with respect to *X*. *P*(***x***|***y***) denotes the conditional probability *P*(*X*=***x***|*Y*=***y***). ***θ*** represents generic parameters of a probability measure, for instance *P*(*X*|*Y*;***θ***) (or *P*_***θ***_(*X*|*Y*)) is the conditional probability parameterized by ***θ***. ***γ*** represents generic hyperparameter vectors. *π*(***θ***;***γ***) is the probability measure over the parameters ***θ*** governed by hyperparameters ***γ***, the parameters themselves governing another probability measure over some random variables. Throughout the paper, the terms “pathway” and “network” are used interchangeably. Also, the terms “feature”’ and “variable” are used interchangeably. $\mathcal {M}ult(\boldsymbol {p};n)$ and $\mathcal {D}(\boldsymbol {\alpha })$ represent a multinomial distribution with vector parameter ***p*** and *n* samples, and a Dirichlet distribution with vector ***α***, respectively.

### Review of optimal Bayesian classification

Binary classification involves a feature vector **X**=(*X*_1_,*X*_2_,…,*X*_*d*_)^*T*^∈ℜ^*d*^ composed of random variables (features), a binary random variable (label) *Y* and a *classifier* *ψ*(**X**) to predict *Y*. The error is *ε*[*ψ*]=*P*(*ψ*(**X**)≠*Y*). An optimal classifier, *ψ*_bay_, called a *Bayes classifier*, has minimal error, called the *Bayes error*, among all possible classifiers. The underlying probability model for classification is the joint feature-label distribution. It determines the class prior probabilities *c*_0_=*c*=*P*(*Y*=0) and *c*_1_=1−*c*=*P*(*Y*=1), and the class-conditional densities *f*_0_(**x**)=*P*(**x**|*Y*=0) and *f*_1_(**x**)=*P*(**x**|*Y*=1). A Bayes classifier is given by

1 $$ \psi_{\text{bay}}(\mathbf{x})\,=\, \left\{\begin{array}{ll} 1, & c_{1}f_{1\!}(\mathbf{x})\geq c_{0}f_{0}(\mathbf{x}), \\ 0, & \text{otherwise.} \end{array}\right. {.}  $$

If the feature-label distribution is unknown but belongs to an uncertainty class of feature-label distributions parameterized by the vector ***θ***∈***Θ***, then, given a random sample *S*_*n*_, an *optimal Bayeisan classifier* (OBC) minimizes the expected error over ***Θ***:

2 $$ \psi_{\text{OBC}}=\arg \min_{\psi \in \mathcal{C}}E_{\pi^{\ast }(\theta)}[\varepsilon_{\boldsymbol{\theta}}[\psi ]],  $$

where the expectation is relative to the posterior distribution *π* ^∗^(***θ***) over ***Θ***, which is derived from the prior distribution *π*(***θ***) using Bayes’ rule [40, 41]. If we let ***θ***_0_ and ***θ***_1_ denote the class 0 and class 1 parameters, then we can write ***θ*** as ***θ***=[*c*,***θ***_***0***_,***θ***_***1***_]. If we assume that *c*,***θ***_0_,***θ***_1_ are independent prior to observing the data, i.e. *π*(***θ***)=*π*(*c*)*π*(***θ***_0_)*π*(***θ***_1_), then the independence is preserved in the posterior distribution *π* ^∗^(***θ***)=*π* ^∗^(*c*)*π* ^∗^(***θ***_0_)*π* ^∗^(***θ***_1_) and the posteriors are given by $\pi ^{\ast }(\boldsymbol {\theta }_{y})\propto \pi (\boldsymbol {\theta } _{y})\prod _{i=1}^{n_{y}}f_{\boldsymbol {\theta }_{y}}(\mathbf {x}_{i}^{y}|y)$ for *y*=0,1, where $f_{\boldsymbol {\theta }_{y}}(\mathbf {x}_{i}^{y}|y)$ and *n*_*y*_ are the class-conditional density and number of sample points for class *y*, respectively [42].

Given a classifier *ψ*_*n*_ designed from random sample *S*_*n*_, from the perspective of mean-square error, the best error estimate minimizes the MSE between its true error (a function of ***θ*** and *ψ*_*n*_) and an error estimate (a function of *S*_*n*_ and *ψ*_*n*_). This Bayesian minimum-mean-square-error (MMSE) estimate is given by the expected true error, $\widehat {\varepsilon }(\psi _{n},S_{n})=\mathrm {E}_{\boldsymbol {\theta } }[\varepsilon (\psi _{n},\boldsymbol {\theta })|S_{n}]$, where *ε*(*ψ*_*n*_,***θ***) is the error of *ψ*_*n*_ on the feature-label distribution parameterized by ***θ*** and the expectation is taken relative to the prior distribution *π*(***θ***) [42]. The expectation given the sample is over the posterior probability. Thus, $\widehat {\varepsilon }(\psi _{n},S_{n})=\mathrm {E}_{\pi ^{\ast }}[\varepsilon ]$.

The *effective class-conditional density* for class *y* is defined by

3 $$ f_{\boldsymbol{\Theta}}\left(\mathbf{x}|y\right) =\int_{\boldsymbol{\Theta}_{y}}f_{\boldsymbol{\theta} _{y}}\left(\mathbf{x}|y\right) \pi^{\ast }\left(\boldsymbol{\theta}_{y}\right) d\boldsymbol{\theta}_{y},  $$

***Θ***_*y*_ being the space for ***θ***_*y*_, and an OBC is given pointwise by [40]

4 $$ \begin{aligned} &\psi_{\text{OBC}}\left(\mathbf{x}\right) = \\ &\left\{ \begin{array}{ll} 0 & \text{if }\ \mathrm{E}_{\pi^{\ast }}[c]f_{\boldsymbol{\Theta}}\left(\mathbf{x} |0\right) \geq (1-\mathrm{E}_{\pi^{\ast }}[c])f_{\boldsymbol{\Theta}}\left(\mathbf{x} |1\right), \\ 1 & \text{otherwise}. \end{array} \right.. \end{aligned}  $$

For discrete classification there is no loss in generality in assuming a single feature *X* taking values in the set {1,…,*b*} of “bins”. Classification is determined by the class 0 prior probability *c* and the class-conditional probability mass functions *p*_*i*_=*P*(*X*=*i*|*Y*=0) and *q*_*i*_=*P*(*X*=*i*|*Y*=1), for *i*=1,…,*b*. With uncertainty, we assume beta class priors and define the parameters ***θ***_0_={*p*_1_,*p*_2_,…,*p*_*b*−1_} and ***θ***_1_={*q*_1_,*q*_2_,…,*q*_*b*−1_}. The bin probabilities must be valid. Thus, {*p*_1_,*p*_2_,…,*p*_*b*−1_}∈***Θ***_0_ if and only if 0≤*p*_*i*_≤1 for *i*=1,…,*b*−1 and $\sum _{i=1}^{b-1}p_{i} \leq 1$, in which case, $p_{b}=1-\sum _{i=1}^{b-1}p_{i}$. We use the Dirichlet priors

5 $$ \pi (\boldsymbol{\theta}_{0})\propto \prod_{i=1}^{b}p_{i}^{\alpha_{i}^{0}-1}\mathrm{\ and\ }\pi (\theta_{1})\propto \prod_{i=1}^{b}q_{i}^{\alpha_{i}^{1}-1}, \mathrm{\ }  $$

where $\alpha _{i}^{y}>0$. These are conjugate priors, leading to the posteriors of the same form. The effective class-conditional densities are

6 $$ f_{\boldsymbol{\Theta}}\left(j|y\right) =\frac{U_{j}^{y}+\alpha_{j}^{y}}{n_{y}+\sum_{i=1}^{b} \alpha_{i}^{y}},   $$

for *y*=0,1, and the OBC is given by

7 $$ {\begin{aligned} \psi_{\text{OBC}}(\,j)=\left\{ \begin{array}{ll} 0,\quad\quad~\text{if }\ E_{\pi^{\ast }}[c]f_{\boldsymbol{\Theta}}\left(j|0\right)\geq (1-E_{\pi^{\ast }}[c])f_{\boldsymbol{\Theta}}\left(j|1\right); \\ 1,\quad\quad~\text{otherwise.} \end{array}\right. \end{aligned}}  $$

where $U_{j}^{y}$ denotes the observed count for class *y* in bin *j* [40]. Hereafter, $\sum _{i=1}^{b}\alpha _{i}^{y}$ is represented by $\alpha _{0}^{y}$, i.e. $\alpha _{0}^{y} = \sum _{i=1}^{b}\alpha _{i}^{y}$, and is called the precision factor. In the sequel, the sub(super)-script relating to dependency on class *y* may be dropped; nonetheless, availability of prior knowledge for both classes is assumed.

### Multinomial mixture model

In practice, data may not be labeled, due to potential tumor-tissue sample or stage heterogeneity, but still we want to classify a new sample point. A mixture model is a natural model for this scenario, assuming each sample point **x**_*i*_ arises from a mixture of multinomial distributions:

8 $$ P_{\boldsymbol{\theta}}(\mathbf{x}_{i}) = \sum_{j=0}^{M-1}c_{j} P_{\boldsymbol{\theta}_{j}}(\mathbf{x}_{i}),  $$

where *M* is the number of components. When there exists two components, similar to binary classification, *M*=2. The conjugate prior distribution family for component probabilities (if unknown) is the Dirichlet distribution. In the mixture model, no closed-form analytical posterior distribution for the parameters exists, but Markov chain Monte Carlo (MCMC) methods [43] can be employed to numerically calculate the posterior distributions. Since the conditional distributions can be calculated analytically in the multinomial mixture model, Gibbs sampling [44, 45] can be employed for the Bayesian inference. If the prior probability distribution over the component probability vector (***c***=[*c*_0_,*c*_1_,…,*c*_*M*_]) is a Dirichlet distribution $\mathcal {D}(\boldsymbol {\phi })$ with parameter vector ***ϕ***, the component-conditional probabilities are $\boldsymbol {\theta }_{j}=[ p^{j}_{1},p^{j}_{2},\ldots,p^{j}_{b}]$, and the prior probability distribution over them is Dirichlet $\mathcal {D}(\boldsymbol {\alpha ^{j}})$ with parameter vector ***α*** ^***j***^ (as in the classification problem), for *j*=1,…,*M*, the Gibbs updates are

$$\begin{aligned} & y_{i}^{(t)} \sim P\left(y_{i}=j|\boldsymbol{c}^{(t-1)},\boldsymbol{\theta}^{(t-1)},\mathbf{x}_{i}\right) \propto c_{j}^{(t-1)} {p_{\mathbf{x}_{i}}^{j,(t-1)}}\\ & \boldsymbol{c}^{(t)} \sim P\left(\boldsymbol{c}|\boldsymbol{\phi},\boldsymbol{y}^{(t)}\right) = \mathcal{D}\left(\boldsymbol{\phi}+ {\sum\nolimits}_{i=1}^{n}\left[I_{y_{i}^{(t)}=1},\ldots,I_{y_{i}^{(t)}=M}\right]\right)\\ & \boldsymbol{\theta_{j}}^{(t)} \sim P\left(\boldsymbol{\theta}_{j}|\mathbf{x},\boldsymbol{y}^{(t)},\boldsymbol{\alpha}_{j}\right)\\ &\quad= \mathcal{D}\left(\boldsymbol{\alpha}_{j}+ {\sum\nolimits}_{i=1:y_{i}^{(t)}=j}^{n}\left[I_{\mathbf{x}_{i}=1},\ldots,I_{\mathbf{x}_{i}=b}\right]\right), \end{aligned} $$

where the super-script in parentheses denotes the chain iteration number, *I*_*w*_ is one if *w* is true, and otherwise *I*_*w*_ is zero. In this framework, if the inference chain runs for *Is* iterations, then the numerical approximation of the OBC classification rule is

9 $$ \psi_{\text{OBC}}(k)\approx \arg \!\max_{y\in { \{1,\dots,M\}}}\sum_{t=1}^{Is}c_{y}^{(t)}p^{y,(t)}_{k}.  $$

Without loss of generality the summation above can be over the iterations of the chain considering burn-in and thinning.

### Prior construction: general framework

In this section, we propose a general framework for prior construction. We begin with introducing a knowledge-driven prior probability:

#### **Definition 1**

(Maximal Knowledge-driven Information Prior) If *Π* is a family of proper priors, then a maximal knowledge-driven information prior (MKDIP) is a solution to the following optimization problem:

10 $$ \arg\min_{\pi \in \Pi}E_{\pi}[C_{\boldsymbol{\theta}}(\xi,D)],  $$

where *C*_***θ***_(*ξ*,*D*)is a cost function that depends on *(1)* ***θ***: the random vector parameterizing the underlying probability distribution, *(2)* *ξ*: state of (prior) knowledge, and *(3)* *D*: partial observation (part of the sample data).

Alternatively, by parameterizing the prior probability as *π*(***θ***;***γ***), with ***γ***∈*Γ* denoting the hyperparameters, an MKDIP can be found by solving

11 $$ \arg\min_{\boldsymbol{\gamma}\in \Gamma}E_{\pi(\boldsymbol{\theta};\boldsymbol{\gamma})}[C_{\boldsymbol{\theta}}(\xi,D,\boldsymbol{\gamma})].  $$

In contrast to non-informative priors, the MKDIP incorporates available prior knowledge and even *part* of the data to construct an informative prior.

The MKDIP definition is very general because we want a general framework for prior construction. The next definition specializes it to cost functions of a specific form in a constrained optimization.

#### **Definition 2**

(MKDIP: Constrained Optimization with Additive Costs) As a special case in which *C*_***θ***_ can be decomposed into additive terms, the cost function is of the form:

$$C_{\boldsymbol{\theta}}(\xi,D,\boldsymbol{\gamma}) =(1-\beta)g^{(1)}_{\boldsymbol{\theta}}(\xi,\boldsymbol{\gamma}) + \beta g^{(2)}_{\boldsymbol{\theta}}(\xi,D), $$

where *β* is a non-negative regularization parameter. In this case, the MKDIP construction with additive costs and constraints involves solving the following optimization problem:

12 $$ \begin{aligned} &\arg\min_{\boldsymbol{\gamma}\in\Gamma}E_{\pi(\boldsymbol{\theta};\boldsymbol{\gamma})} \Big[(1-\beta)g^{(1)}_{\boldsymbol{\theta}}(\xi,\boldsymbol{\gamma}) + \beta g^{(2)}_{\boldsymbol{\theta}}(\xi,D)\Big]\\ & \text{Subject to:} \quad E_{\pi(\boldsymbol{\theta};\boldsymbol{\gamma})}[g^{(3)}_{\boldsymbol{\theta},i}(\xi)]=0;~i\in\{1,\ldots,n_{c}\}, \end{aligned}  $$

where $g^{(3)}_{\boldsymbol {\theta },i}$, ∀*i*∈{1,…,*n*_*c*_}, are constraints resulting from the state of knowledge *ξ*via a mapping:

$$\mathcal{T}: \xi \rightarrow E_{\pi\left(\boldsymbol{\theta};\boldsymbol{\gamma}\right)}\left[g^{(3)}_{\boldsymbol{\theta},i}(\xi)\right], \forall i \in \{1,\dots,n_{c}\}. $$

In the sequel, we will refer to *g* ^(1)^(·) and *g* ^(2)^(·) as the cost functions, and $g_{i}^{(3)}(\cdot)$’s as the knowledge-driven constraints. We begin with introducing information-theoretic cost functions, and then we propose a general set of mapping rules, denoted by $\mathcal {T}$ in Definition 2, to convert biological pathway knowledge into mathematical forms. We then consider special cases with information-theoretic cost functions.

### Information-theoretic cost functions

Instead of having least squares (or mean-squared error) as the standard cost functions in classical statistical inference problems, there is no universal cost function in the prior construction literature. That being said, in this paper, we utilize several widely used cost functions in the field:

1. (Maximum Entropy) The principle of maximum-entropy (MaxEnt) for probability construction [38] leads to the least informative prior given the constraints in order to prevent adding spurious information. Under our general framework MaxEnt can be formulated by setting:
  $$\beta=0,~g_{\boldsymbol{\theta}}^{(1)} = -H[\boldsymbol{\theta}], $$
  where *H*[.] denotes the Shannon entropy.
2. (Maximal Data Information) The maximal data information prior (MDIP) introduced by Zellner [46] as a choice of the objective function is a criterion for the constructed probability distribution to remain maximally committed to the data [47]. To achieve MDIP, we can set our general framework with:
  $$\begin{aligned} \beta=0,~g_{\boldsymbol{\theta}}^{(1)} &= \ln \pi(\boldsymbol{\theta};\boldsymbol{\gamma}) + H[P(x|\boldsymbol{\theta})]\\ &= \ln \pi(\boldsymbol{\theta};\boldsymbol{\gamma})-E_{x|\boldsymbol{\theta}}[\ln P(x|\boldsymbol{\theta})]. \end{aligned} $$
3. (Expected Mean Log-likelihood) The cost function introduced in [35] is the first one that utilizes part of the observed data for prior construction. In that, we have
  $$\beta =1,~g^{(2)}_{\boldsymbol{\theta}}=-\ell(\boldsymbol{\theta};D), $$
  where $\ell (\boldsymbol {\theta };D)=\frac {1}{n_{D}}\sum _{i=1}^{n_{D}}\log f(\boldsymbol {x}_{i}|\boldsymbol {\theta })$ is the mean log-likelihood function of the sample points used for prior construction (*D*), and *n*_*D*_ denotes the number of sample points in *D*. In [35], it is shown that this cost function is equivalent to the average Kullback-Leibler distance between the *unknown* distribution (empirically estimated by some part of the samples) and the uncertainty class of distributions.

As originally proposed, the preceding approaches did not involve expectation over the uncertainty class. They were extended to the general prior construction form in Definition 1, including the expectation, in [36] to produce the regularized maximum entropy prior (RMEP), the regularized maximal data information prior (RMDIP), and the regularized expected mean log-likelihood prior (REMLP). In all cases, optimization was subject to specialized constraints.

For MKDIP, we employ the same information-theoretic cost functions in the prior construction optimization framework. MKDIP-E, MKDIP-D, and MKDIP-R correspond to using the same cost functions as REMP, RMDIP, and REMLP, respectively, but with the new general types of constraints. To wit, we employ *functional information* from the signaling pathways and show that by adding these new constraints that can be readily derived from prior knowledge, we can improve both supervised (classification problem with labelled data) and unsupervised (mixture problem without labels) learning of Bayesian operators.

### From prior knowledge to mathematical constraints

In this part, we present a general formulation for mapping the existing knowledge into a set of *constraints*. In most scientific problems, the prior knowledge is in the form of conditional probabilities. In the following, we consider a hypothetical gene network and show how each component in a given network can be converted into the corresponding inequalities as general constraints in MKDIP optimization.

Before proceeding we would like to say something about contextual effects on regulation. Because a regulatory model is not independent of cellular activity outside the model, complete control relations such as *A*→*B* in the model, meaning that gene *B* is up-regulated if and only if gene *A* is up-regulated (after some time delay), do not necessarily translate into conditional probability statements of the form *P*(*X*_*B*_=1|*X*_*A*_=1)=1, where *X*_*A*_ and *X*_*B*_ represent the binary gene values corresponding to genes *A* and *B*, respectively. Rather, what may be observed is *P*(*X*_*B*_=1|*X*_*A*_=1)=1−*δ*, where *δ*>0. The pathway *A*→*B* need not imply *P*(*X*_*B*_=1|*X*_*A*_=1)=1 because *A*→*B* is conditioned on the *context* of the cell, where by context we mean the overall state of the cell, not simply the activity being modeled. *δ* is called a *conditioning* parameter. In an analogous fashion, rather than *P*(*X*_*B*_=1|*X*_*A*_=0)=0, what may be observed is *P*(*X*_*B*_=1|*X*_*A*_=0)=*η*, where *η*>0, because there may be regulatory relations outside the model that up-regulate *B*. Such activity is referred to as cross-talk and *η* is called a *crosstalk* parameter. Conditioning and cross-talk effects can involve multiple genes and can be characterized analytically via context-dependent conditional probabilities [48].

Consider binary gene values *X*_1_,*X*_2_,…,*X*_*m*_ corresponding to genes *g*_1_,*g*_2_,…,*g*_*m*_. There are *m*2^*m*−1^ conditional probabilities of the form

13 $$\begin{array}{@{}rcl@{}} &&P(X_{i}=k_{i}| X_{1}=k_{1},\dots,X_{i-1}=k_{i-1},X_{i+1}=  \\ &&\quad k_{i+1},\dots,X_{m}=k_{m})\\ &&=a^{k_{i}}_{i}(k_{1},\dots,k_{i-1},k_{i+1},\dots,k_{m}) \end{array} $$

to serve as constraints, the chosen constraints to be the conditional probabilities whose values are known (approximately). For instance, if *g*_2_ and *g*_3_ regulate *g*_1_, with *X*_1_=1 when *X*_2_=1 and *X*_3_=0, then, ignoring context effects,

$$a^{1}_{1}(1,0,k_{4},\dots,k_{m})=1 $$

for all *k*_4_,…,*k*_*m*_. If, however, we take context conditioning into effect, then

$$a_{1}^{1}(1,0,k_{4},\dots,k_{m}) = 1-\delta_{1}(1,0,k_{4},\dots,k_{m}), $$

where *δ*_1_(1,0,*k*_4_,…,*k*_*m*_) is a conditioning parameter.

Moreover, ignoring context effects,

$$\begin{array}{@{}rcl@{}} a^{1}_{1}(1,1,k_{4},\dots,k_{m})&=& a^{1}_{1}(0,0,k_{4},\dots,k_{m})\\ &=&a^{1}_{1}(0,1,k_{4},\dots,k_{m}) = 0 \end{array} $$

for all *k*_4_,…,*k*_*m*_. If, however, we take crosstalk into effect, then

$$\begin{array}{@{}rcl@{}} a^{1}_{1}(1, 1, k_{4},\dots, k_{m})& =& \eta_{1}(1, 1, k_{4},\dots, k_{m})\\ a^{1}_{1}(0, 0, k_{4},\dots, k_{m}) &=& \eta_{1}(0, 0, k_{4},\dots, k_{m})\\ a^{1}_{1}(0, 1, k_{4},\dots, k_{m}) &=& \eta_{1}(0, 1, k_{4},\dots, k_{m}), \end{array} $$

where *η*_1_(1,1,*k*_4_,…,*k*_*m*_), *η*_1_(0,0,*k*_4_,…,*k*_*m*_), and *η*_1_(0,0,*k*_4_,…,*k*_*m*_) are crosstalk parameters. In practice it is unlikely that we would know the conditioning and crosstalk parameters for all combinations of *k*_4_,…,*k*_*m*_; rather, we might just know the average, in which case, *δ*_1_(1,0,*k*_4_,…,*k*_*m*_) reduces to *δ*_1_(1,0), *η*_1_(1,1,*k*_4_,…,*k*_*m*_) reduces to *η*_1_(1,1), etc.

In this paradigm, the constraints resulting from our state of knowledge are of the following form:

14 $$ \begin{aligned} &g^{(3)}_{\boldsymbol{\theta},i}(\xi)=\\ &P(X_{i}=k_{i}| X_{1}=k_{1},\dots,X_{i-1}=k_{i-1},X_{i+1}=k_{i+1}, \\ & \quad \dots,X_{m}=k_{m}) -a^{k_{i}}_{i}(k_{1},\dots,k_{i-1},k_{i+1},\dots,k_{m}). \end{aligned}  $$

The basic setting is very general and the conditional probabilities are what they are, whether or not they can be expressed in the regulatory form of conditioning or crosstalk parameters. The general scheme includes previous constraints and approaches proposed in [35] and [36] for the Gaussian and discrete setups. Moreover, in those we can drop the regulatory-set entropy because it is replaced by the set of conditional probabilities based on the regulatory set, whether forward (master predicting slaves) or backwards (slaves predicting masters) [48].

In this paradigm, the optimization constraints take the form

15 $$\begin{array}{@{}rcl@{}} && a^{k_{i}}_{i}(k_{1},\dots, k_{i-1}, k_{i+1},\dots, k_{m})-  \\ && \quad \varepsilon_{i}(k_{1},\dots, k_{i-1}, k_{i+1},\dots, k_{m})  \\ && \leq E_{\pi(\boldsymbol{\theta};\boldsymbol{\gamma})}[P(X_{i} = k_{i}| X_{1} = k_{1},\dots, X_{i-1} = k_{i-1}, \\ && \quad \quad \quad \quad X_{i+1} = k_{i+1},\dots, X_{m} = k_{m})] \\ && \leq a^{k_{i}}_{i}(k_{1},\dots, k_{i-1}, k_{i+1},\dots, k_{m}) +  \\ &&\quad \quad \varepsilon_{i}(k_{1},\dots, k_{i-1}, k_{i+1},\dots, k_{m}), \end{array} $$

where the expectation is with respect to the uncertainty in the model parameters, that is, the distribution of the model parameter ***θ***, and *ε*_*i*_ is a slackness variable. Not all will be used, depending on our prior knowledge. In fact, the general conditional probabilities will not likely be used because they will likely not be known when there are too many conditioning variables. For instance, we may not know the probability in Eq. (13), but may know the conditioning on part of the variables which can be extracted from some interaction network (e.g. biological pathways). A slackness variable can be considered for each constraint to make the constraint framework more flexible, thereby allowing potential error or uncertainty in prior knowledge (allowing potential inconsistencies in prior knowledge). When using slackness variables, these variables also become optimization parameters, and a linear function (summation of all slackness variables) times a regulatory coefficient is added to the cost function of the optimization in Eq. (12). In other words, when having slackness variables, the optimization in Eq. (12) can be written as

16 $$  \begin{aligned} & \arg\min_{\boldsymbol{\gamma}\in\Gamma, \boldsymbol{\varepsilon}\in\mathcal{E}}E_{\pi(\boldsymbol{\theta};\boldsymbol{\gamma})}\Big[\lambda_{1}[(1-\beta)g^{(1)}_{\boldsymbol{\theta}}(\xi,\boldsymbol{\gamma}) + \beta g^{(2)}_{\boldsymbol{\theta}}(\xi,D)] \\ & \quad \quad \quad \quad \quad \quad \quad \quad \quad + \lambda_{2}\sum_{i=1}^{n_{c}}\varepsilon_{i}\Big]\\ & \text{Subject to:} -\varepsilon_{i}\leq E_{\pi(\boldsymbol{\theta};\boldsymbol{\gamma})}[g^{(3)}_{\boldsymbol{\theta},i}(\xi)]\leq\varepsilon_{i};~i\in\{1,\ldots,n_{c}\}, \end{aligned}  $$

where *λ*_1_ and *λ*_2_ are non-negative regularization parameters, and ***ε*** and $\mathcal {E}$ represent the vector of all slackness variables and the feasible region for slackness variables, respectively. For each slackness variable, a possible range can be defined (note that all slackness variables are non-negative). The higher the uncertainty is about a constraint stemming from prior knowledge, the greater the possible range for the corresponding slackness variable can be (more on this in the “Results and discussion” section).

The new general type of constraints discussed here introduces a formal procedure for incorporating prior knowledge. It allows the incorporation of knowledge of the functional regulations in the signaling pathways, any constraints on the conditional probabilities, and also knowledge of the cross-talk and conditioning parameters (if present), unlike the previous work in [36] where only partial information contained in the edges of the pathways is used in an ad hoc way.

### An illustrative example and connection with conditional entropy

**Fig. 2:**
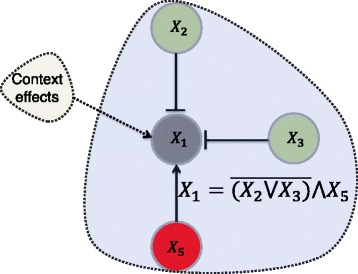
An illustrative example showing the components directly connected to gene 1. In the Boolean function {AND, OR, NOT}={∧,∨,−}. Based on the regulating function of gene 1, it is up-regulated if gene 5 is up-regulated and genes 2 and 3 are down-regulated

Now, consider a hypothetical network depicted in Fig. 2. For instance, suppose we know that the expression of gene *g*_1_ is regulated by *g*_2_, *g*_3_, and *g*_5_. Then we have

$$P(X_{1} = 1| X_{2} = k_{2}, X_{3} = k_{3}, X_{5} = k_{5}) = a_{1}^{1}(k_{2}, k_{3}, k_{5}).$$

As an example,

$$P(X_{1} = 1| X_{2} = 1, X_{3} = 1, X_{5} = 0) = a_{1}^{1}(1_{2}, 1_{3}, 0_{5}), $$

where the notation 1_2_ denotes 1 for the second gene. Further, we might not know *a*_1_(*k*_2_,*k*_3_,*k*_5_) for all combinations of *k*_2_, *k*_3_, *k*_5_. Then we use the ones that we know. In the case of conditioning with *g*_2_, *g*_3_, and *g*_5_ regulating *g*_1_, with *g*_1_ on if the others are on,

$$a^{1}_{1}(1_{2}, 1_{3}, 1_{5}) = 1 -\delta_{1}(1_{2}, 1_{3}, 1_{5}). $$

If limiting to 3-gene predictors, *g*_3_, and *g*_5_ regulate *g*_1_, with *g*_1_ on if the other two are on, then

$$a^{1}_{1}(k_{2}, 1_{3}, 1_{5}) = 1 - \delta_{1}(k_{2}, 1_{3}, 1_{5}), $$

meaning that the conditioning parameter depends on whether *X*_2_=0 or 1.

Now, considering the conditional entropy, assuming that $\delta _{1} = \max _{(k_{2},k_{3},k_{5})} \delta _{1}(k_{2},k_{3},k_{5})$ and *δ*_1_<0.5, we may write

$$ \begin{aligned} &H[X_{1}|X_{2},X_{3},X_{5}] =\\ &-\left[\sum_{\mathcal{X}_{2},\mathcal{X}_{3},\mathcal{X}_{5}}\left[ P\left(X_{1}=0|X_{2}=x_{2},X_{3}=x_{3},X_{5}=x_{5}\right)\right.\right.\\ &\quad \quad\times P\left(X_{2}=x_{2},X_{3}=x_{3},X_{5}=x_{5}\right)\\ &\quad \quad\log\left[P\left(X_{1}=0|X_{2}=x_{2},X_{3}=x_{3},X_{5}=x_{5}\right)\right]\\ &\quad \quad +P\left(X_{1}=1|X_{2}=x_{2},X_{3}=x_{3},X_{5}=x_{5}\right)\\ &\quad \quad\times P\left(X_{2}=x_{2},X_{3}=x_{3},X_{5}=x_{5}\right)\\ &\quad \quad\left.\left.\log\left[P\left(X_{1}=1|X_{2}=x_{2}, X_{3}=x_{3},X_{5}=x_{5}\right)\right]\right]{\vphantom{\sum_{\mathcal{X}_{2}}}}\right]\\ &\quad\quad\quad\leq h(\delta_{1}), \end{aligned} $$

where *h*(*δ*)=−[*δ* log(*δ*)+(1−*δ*) log(1−*δ*)]. Hence, bounding the conditional probabilities, the conditional entropy is in turn bounded by *h*(*δ*_1_):

$${\lim}_{\delta_{1}\rightarrow 0^{+}} H\left[X_{1}|X_{2},X_{3},X_{5}\right] = 0. $$

It should be noted that constraining *H*[*X*_1_|*X*_2_,*X*_3_,*X*_5_] would not necessarily constrain the conditional probabilities, and may be considered as a more relaxed type of constraints. But, for example, in cases where there is no knowledge about the status of a gene given its regulator genes, constraining entropy is the only possible approach.

In our illustrative example, if we assume that the Boolean regulating function of *X*_1_ is known as shown in Fig. 2 and context effects exist, then the following knowledge constraints can be extracted from the pathway and regulating function:

$$\begin{aligned} &a^{0}_{1}\left(k_{2}, k_{3}, 0_{5}\right) = 1 - \delta_{1}\left(k_{2}, k_{3}, 0_{5}\right)\\ &a^{0}_{1}\left(k_{2}, 1_{3}, k_{5}\right) = 1 - \delta_{1}\left(k_{2}, 1_{3}, k_{5}\right)\\ &a^{0}_{1}\left(1_{2}, k_{3}, k_{5}\right) = 1 - \delta_{1}\left(1_{2}, k_{3}, k_{5}\right)\\ &a^{1}_{1}\left(0_{2}, 0_{3}, 1_{5}\right) = 1 - \delta_{1}\left(0_{2}, 0_{3}, 1_{5}\right). \end{aligned} $$

Now if we assume that the context does not affect the value of *X*_1_, i.e. the value of *X*_1_ can be fully determined by knowing the values of *X*_2_, *X*_3_, and *X*_5_, then we have the following equations:

17a $$\begin{array}{*{20}l} & a^{0}_{1}\left(k_{2}, k_{3}, 0_{5}\right) = P\left(X_{1}=0|X_{5}=0\right) = 1 \end{array} $$

17b $$\begin{array}{*{20}l} & a^{0}_{1}\left(k_{2}, 1_{3}, k_{5}\right) = P\left(X_{1}=0|X_{3}=1\right) = 1 \end{array} $$

17c $$\begin{array}{*{20}l} & a^{0}_{1}\left(1_{2}, k_{3}, k_{5}\right) = P\left(X_{1}=0|X_{2}=1\right)= 1 \end{array} $$

17d $$\begin{array}{*{20}l} & a^{1}_{1}\left(0_{2}, 0_{3}, 1_{5}\right)= P(X_{1}=1|X_{2}=0,X_{3}=0,  \\ & \quad \quad \quad \quad \quad \quad \quad \quad \quad \quad \quad \quad \quad X_{5}=1) = 1. \end{array} $$

It can be seen from the equations above that for some setups of the regulator values, only a subset of them determines the value of *X*_1_, regardless of the other regulator values. If we assume that the value of *X*_5_ cannot be observed, for example *X*_5_ is an extracellular signal that cannot be measured in gene expression data and thereafter *X*_5_ is not in the features of our data, the only constraints relevant to the feature-label distribution that can be extracted from the regulating function knowledge will be

18 $$ \begin{aligned} &a^{0}_{1}\left(k_{2}, 1_{3}, k_{5}\right) = P\left(X_{1}=0|X_{3}=1\right) = 1\\ & a^{0}_{1}\left(1_{2}, k_{3}, k_{5}\right) = P\left(X_{1}=0|X_{2}=1\right)= 1. \end{aligned}  $$

### Special case of Dirichlet distribution

Fixing the value of a single gene, being ON or OFF (i.e. *X*_*i*_=0 or *X*_*i*_=1, respectively), corresponds to a partition of the states, $\mathcal {X }=\{1,\dots,b\}$. Here, the portions of $\mathcal {X}$ for which (*X*_*i*_=*k*_1_,*X*_*j*_=*k*_2_) and (*X*_*i*_≠*k*_1_,*X*_*j*_=*k*_2_), for any *k*_1_,*k*_2_∈{0,1}, are denoted by $\mathcal {X}^{i,j}(k_{1},k_{2})$ and $\mathcal {X}^{i,j}(k_{1}^{c},k_{2})$, respectively. For the Dirichlet distribution, where ***θ***=***p*** and ***γ***=***α***, the constraints on the expectation over the conditional probability in (15) can be explicitly written as functions of the prior probability parameters (hyperparameters). For the parameter of the Dirichlet distribution, a vector ***α*** indexed by $\mathcal {X}$, we denote the variable indicating the summation of its entities in $\mathcal { X}^{i,j}(k_{1},k_{2})$ by $\overline {\alpha }^{i,j}(k_{1},k_{2})=\sum _{k \in \mathcal {X}^{i,j}(k_{1},k_{2})}\alpha _{k}$. The notation can be easily extended for the cases having more than two fixed genes. In this setup, if the set of random variables corresponding to genes other than *g*_*i*_ and the vector of their corresponding values are shown by $\tilde {X}_{i}$ and $\tilde {X}_{i}$, respectively, the expectation over the conditional probability in (15) is [36]:

19 $$ \hspace{12pt} \begin{aligned} &E_{\boldsymbol{p}}\left[P\left(X_{i} = k_{i}| X_{1} = k_{1},\dots, X_{i-1} = k_{i-1},\right.\right.\\ &\quad\left. \left.X_{i+1} = k_{i+1},\dots, X_{m} = k_{m}\right)\right]\\ & =E_{\boldsymbol{p}}\left[\frac{\sum_{k\in \mathcal{X}^{i,\tilde{X}_{i}}\left(k_{i},\tilde{x}_{i}\right)}p_{k}}{\sum_{k\in \mathcal{X}^{i,\tilde{X}_{i}}\left(k_{i},\tilde{x}_{i}\right)}p_{k}+\sum_{k\in \mathcal{X}^{i,\tilde{X}_{i}}\left(k_{i}^{c},\tilde{x}_{i}\right)}p_{k}}\right] \\ &=\frac{\overline{\alpha }^{i,\tilde{X}_{i}}\left(k_{i},\tilde{x}_{i}\right)}{\overline{\alpha }^{i,\tilde{X}_{i}}\left(k_{i},\tilde{x}_{i}\right)+\overline{\alpha }^{i,\tilde{X}_{i}}\left(k_{i}^{c},\tilde{x}_{i}\right)}, \end{aligned}  $$

where the summation in the numerator and the first summation in the denominator of the second equality is over the states (bins) for which ($X_{i} = k_{i}, \tilde {X}_{i} = \tilde {x}_{i}$), and the second summation in the denominator is over the states (bins) for which ($X_{i} = k_{i}^{c}, \tilde {X}_{i} = \tilde {x}_{i}$).

If there exists a set of genes that completely determines the value of gene *g*_*i*_ (or only a specific setup of their values that determines the value, as we had in our illustrative example in Eq. (17)), then the constraints on the conditional probability conditioned on all the genes other than *g*_*i*_ can be changed to be conditioned on that set only. Specifically, let ***R***_*i*_ denote the set of random variables corresponding to such a set of genes/proteins and suppose there exists a specific setup of their values ***r***_*i*_ that completely determines the value of gene *g*_*i*_. If the set of all random variables corresponding to the genes/proteins other than *X*_*i*_ and ***R***_*i*_ is denoted by $\boldsymbol {B}_{i}=\tilde {X}_{(i,\boldsymbol {R}_{i})}$, and their corresponding values by ***b***_*i*_, then the constraints on the conditional probability can be written as

20 $$ \begin{aligned} &E_{\boldsymbol{p}}\left[P\left(X_{i} = k_{i}| \boldsymbol{R}_{i} = \boldsymbol{r}_{i}\right)\right]\\ &=E_{\boldsymbol{p}}\left[\frac{\sum_{\boldsymbol{b}_{i}\in O_{\boldsymbol{B}_{i}}}\sum_{k\in \mathcal{X}^{i,\boldsymbol{R}_{i},\boldsymbol{B}_{i}}\left(k_{i},\boldsymbol{r}_{i},\boldsymbol{b}_{i}\right)}p_{k}}{\sum_{\boldsymbol{b}_{i}\in O_{\boldsymbol{B}_{i}}}\sum_{k\in \mathcal{X}^{i,\boldsymbol{R}_{i},\boldsymbol{B}_{i}}(k_{i},\boldsymbol{r}_{i},\boldsymbol{b}_{i})}p_{k}}\right.\\ & \quad \quad \quad\left.\frac{}{+ \sum_{\boldsymbol{b}_{i}\in O_{\boldsymbol{B}_{i}}}\sum_{k\in \mathcal{X}^{i,\boldsymbol{R}_{i},\boldsymbol{B}_{i}}\left(k_{i}^{c},\boldsymbol{r}_{i},\boldsymbol{b}_{i}\right)}p_{k}}\right] \\ &= \frac{\sum_{\boldsymbol{b}_{i}\in O_{\boldsymbol{B}_{i}}}\overline{\alpha }^{i,\boldsymbol{R}_{i},\boldsymbol{B}_{i}}(k_{i},\boldsymbol{r}_{i},\boldsymbol{b}_{i})}{\sum_{\boldsymbol{b}_{i}\in O_{\boldsymbol{B}_{i}}}\overline{\alpha }^{i,\boldsymbol{R}_{i},\boldsymbol{B}_{i}}(k_{i},\boldsymbol{r}_{i},\boldsymbol{b}_{i})} \\ & \quad \quad \frac{}{+\sum_{\boldsymbol{b}_{i}\in O_{\boldsymbol{B}_{i}}}\overline{\alpha }^{i,\boldsymbol{R}_{i},\boldsymbol{B}_{i}}(k_{i}^{c},\boldsymbol{r}_{i},\boldsymbol{b}_{i})}, \end{aligned}   $$

where $\phantom {\dot {i}\!}O_{\boldsymbol {B}_{i}}$ is the set of all possible vectors of values for ***B***_*i*_.

For a multinomial model with a Dirichlet prior distribution, a constraint on the conditional probabilities translates into a constraint on the above expectation over the conditional probabilities (as in Eq. (15)). In our illustrative example and from the equations in Eq. (17), there are four of these constraints on the conditional probability for gene *g*_1_. For example, in the second constraint from the second line of Eq. (17) (Eq. 17b), *X*_*i*_=*X*_1_, *k*_*i*_=0, ***R***_*i*_={*X*_3_}, ***r***_*i*_=[0], and ***B***_*i*_={*X*_2_,*X*_5_}. One might have several constraints for each gene extracted from its regulatory function (more on extracting general constraints from regulating functions in the “Results and discussion” section).

## Results and discussion

The performance of the proposed general prior construction framework with different types of objective functions and constraints is examined and compared with other methods on two pathways, a mammalian cell-cycle pathway and a pathway involving the gene TP53. Here we employ Boolean network modeling of genes/proteins (hereafter referred to as entities or nodes) [49] with perturbation (BNp). A Boolean Network with *p* nodes (genes/proteins) is defined as *B*=(*V*,*F*), where *V* represents the set of entities (genes/proteins) {*v*_1_,…,*v*_*p*_}, and *F* is the set of Boolean predictor functions {*f*_1_,…,*f*_*p*_}. At each step in a BNp, a decision is made by a Bernoulli random variable with the success probability equal to the perturbation probability, *p*_*pert*_, as to whether a node value is determined by perturbation of randomly flipping its value or by the logic model imposed from the interactions in the signaling pathways. A BNp with a positive perturbation probability can be modeled by an ergodic Markov chain, and possesses a steady-state distribution (SSD) [50]. The performance of different prior construction methods can be compared based on the expected true error of the optimal Bayesian classifiers designed with those priors, and also by comparing these errors with some other well known classification methods. Another comparison metric of prior construction methods is the expected norm of the difference between the true parameters and the posterior mean of these parameters inferred using the constructed prior distributions. Here, the true parameters are the vectors of the true class-conditional SSDs, i.e. the vectors of the true class-conditional bin probabilities of the BNp.

Moreover, the performance of the proposed framework is compared with other methods on a publicly available gene expression dataset of non-small cell lung cancer when combined with the existing prior knowledge on relevant signaling pathways.

### Mammalian cell cycle classification

**Fig. 3:**
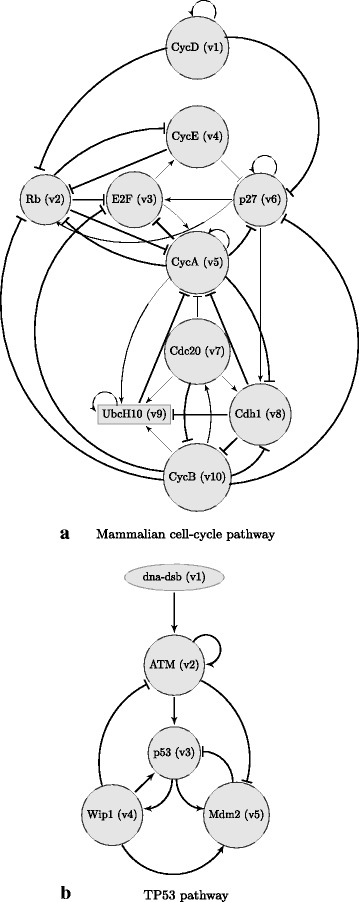
Signaling pathways corresponding to Tables 1 and 2. Signaling pathways for: 3(a) the normal mammalian cell cycle (corresponding to Table 1) and 3(b) a simplified pathway involving TP53 (corresponding to Table 2)

A Boolean logic regulatory network for the dynamical behavior of the cell cycle of normal mammalian cells is proposed in [51]. Figure 3(a) shows the corresponding pathways. In normal cells, cell division is coordinated via extracellular signals controlling the activation of CycD. Rb is a tumor suppressor gene and is expressed when the inhibitor cyclins are not present. Expression of p27 blocks the action of CycE or CycA, and lets the tumor-suppressor gene Rb be expressed even in the presence of CycE and CycA, and results in a stop in the cell cycle. Therefore, in the wild-type cell-cycle network, expressing p27 lets the cell cycle stop. But following the proposed mutation in [51], for the mutated case, p27 is always inactive (i.e. can never be activated), thereby creating a situation where both CycD and Rb might be inactive and the cell can cycle in the absence of any growth factor.

**Table 1:** Boolean regulating functions of normal mammalian cell cycle [51]. In the Boolean functions {AND, OR, NOT}={∧,∨,−}

Gene	Node name	Boolean regulating function
CycD	*v*_1_	Extracellular signal
Rb	*v*_2_	$(\overline {v_{1}}\wedge \overline {v_{4}} \wedge \overline {v_{5}} \wedge \overline {v_{10}})\vee (v_{6}\wedge \overline {v_{1}}\wedge \overline {v_{10}})$
E2F	*v*_3_	$(\overline {v_{2}}\wedge \overline {v_{5}} \wedge \overline {v_{10}})\vee ({v_{6}} \wedge \overline {v_{2}}\wedge \overline {v_{10}})$
CycE	*v*_4_	$({v_{3}}\wedge \overline {v_{2}})$
CycA	*v*_5_	$({v_{3}}\wedge \overline {v_{2}} \wedge \overline {v_{7}} \wedge \overline {(v_{8}\wedge v_{9})})\vee (v_{5}\wedge \overline {v_{2}}\wedge \overline {v_{7}}\wedge $
		$\overline {(v_{8}\wedge v_{9})})$
p27	*v*_6_	$(\overline {v_{1}}\wedge \overline {v_{4}} \wedge \overline {v_{5}} \wedge \overline {v_{10}})\vee (v_{6}\wedge \overline {(v_{4}\wedge v_{5})}\wedge \overline {v_{10}}\wedge \overline {v_{1}})$
Cdc20	*v*_7_	*v*_10_
Cdh1	*v*_8_	$(\overline {v_{5}}\wedge \overline {v_{10}}) \vee ({v_{7}}) \vee {(v_{6}\wedge \overline {v_{10}})}$
UbcH10	*v*_9_	$(\overline {v_{8}})\vee ({v_{8}}\wedge {v_{9}} \wedge ({v_{7}\vee {v_{5}}\vee {v_{10}}}))$
CycB	*v*_10_	$(\overline {v_{7}}\wedge \overline {v_{8}})$

**Table 2:** Boolean regulating functions corresponding to the pathway in Fig. 3(b) [54]. In the Boolean functions {AND, OR, NOT}={∧,∨,−}

Gene	Node name	Boolean regulating function
dna−dsb	*v*_1_	Extracellular signal
ATM	*v*_2_	$\overline {v_{4}} \wedge (v_{2}\vee v_{1})$
P53	*v*_3_	$\overline {v_{5}}\wedge (v_{2}\vee v_{4})$
Wip1	*v*_4_	*v*_3_
Mdm2	*v*_5_	$\overline {v_{2}}\wedge (v_{3}\vee v_{4})$

The full functional regulations in the cell-cycle Boolean network are shown in Table 1.

Following [36], for the binary classification problem, *y*=0 corresponds to the normal system functioning based on Table 1, and *y*=1 corresponds to the mutated (cancerous) system where CycD, p27, and Rb are permanently down-regulated (are stuck at zero), which creates a situation where the cell cycles even in the absence of any growth factor. The perturbation probability is set to 0.01 and 0.05 for the normal and mutated system, respectively. A BNp has a transition probability matrix (TPM), and as mentioned earlier, with positive perturbation probability can be modeled by an ergodic Markov chain, and possesses a SSD [50]. Here, each class has a vector of steady-state bin probabilities, resulting from the regulating functions of its corresponding BNp and the perturbation probability. The constructed SSDs are further marginalized to a subset of seven genes to prevent trivial classification scenarios. The final feature vector is **x**=[E2F,CycE,CycA,Cdc20,Cdh1,UbcH10,CycB], and the state space size is 2^7^=128. The true parameters for each class are the final class-conditional steady-state bin probabilities, ***p*** ^0^ and ***p*** ^1^ for the normal and mutated systems, respectively, which are utilized for taking samples.

### Classification problem corresponding to TP53

TP53 is a tumor suppressor gene involved in various biological pathways [36]. Mutated p53 has been observed in almost half of the common human cancers [52], and in more than 90% of patients with severe ovarian cancer [53]. A simplified pathway involving TP53, based on logic in [54], is shown in Fig. 3(b). DNA double-strand break affects the operation of these pathways, and the Boolean network modeling of these pathways under this uncertainty has been studied in [53, 54]. The full functional regulations are shown in Table 2.

Following [36], two scenarios, dna-dsb=0 and dna-dsb=1, weighted by 0.95 and 0.05, are considered and the SSD of the normal system is constructed based on the ergodic Markov chain model of the BNp with the regulating functions in Table 2 by assuming the perturbation probability 0.01. The SSD for the mutated (cancerous) case is constructed by assuming a permanent down regulation of TP53 in the BNp, and perturbation probability 0.05. Knowing that dna-dsb is not measurable, and to avoid trivial classification situations, the SSDs are marginalized to a subset of three entities **x**=[ATM,Wip1,Mdm2]. The state space size in this case is 2^3^=8. The true parameters for each class are the final class-conditional steady-state bin probabilities, ***p*** ^0^ and ***p*** ^1^ for the normal and mutated systems, respectively, which are used for data generation.

### Extracting general constraints from regulating functions

If knowledge of the regulating functions exists, it can be used in the general constraint framework of the MKDIP, i.e. it can be used to constrain the conditional probabilities. In other words, the knowledge about the regulating function of gene *i* can be used to set *ε*_*i*_(*k*_1_,…,*k*_*i*−1_,*k*_*i*+1_,…,*k*_*m*_), and $a^{k_{i}}_{i}(k_{1},\dots, k_{i-1}, k_{i+1},\dots, k_{m})$ in the general form of constraints in (15). If the true regulating function of gene *i* is known, and it is not context sensitive, then the conditional probability of its status, $a^{k_{i}}_{i}(k_{1},\dots, k_{i-1}, k_{i+1},\dots, k_{m})$, is known for sure, and *δ*_*i*_(*k*_1_,…,*k*_*i*−1_,*k*_*i*+1_,…,*k*_*m*_)=0. But in reality, the true regulating functions are not known, and are also context sensitive. The dependence on the context translates into *δ*_*i*_(*k*_1_,…,*k*_*i*−1_,*k*_*i*+1_,…,*k*_*m*_) being greater than zero. The greater the context effect on the gene status, the larger *δ*_*i*_ is. Moreover, the uncertainty over the regulating function is captured by the slackness variables *ε*_*i*_(*k*_1_,…,*k*_*i*−1_,*k*_*i*+1_,…,*k*_*m*_) in Eq. (15). In other words, the uncertainty is translated to the possible range of the slackness variable values in the prior construction optimization framework. The higher the uncertainty is, the greater the range should be in the optimization framework. In fact, slackness variables make the whole constraint framework consistent, even for cases where the conditional probability constraints imposed by prior knowledge are not completely in line with each other, and guarantee the existence of a solution.

As an example, for the classification problems of the mammalian cell-cycle network and the TP53 network, assuming the regulating functions in Tables 1 and 2 are the true regulating functions, the context effect can be observed in the dependence of the output of the Boolean regulating functions in the tables on the extracellular signals, non-measurable entities, and the genes that have been marginalized out in our setup. In the absence of quantitative knowledge about the context effect, i.e. $a^{k_{i}}_{i}(k_{1},\dots, k_{i-1}, k_{i+1},\dots, k_{m})$ for all possible setups of the regulator values, one can impose only those with such knowledge. For example, in the mammalian cell-cycle network, CycB’s regulating function only depends on the values included in the observed feature set; therefore the conditional probabilities are known for all regulator value setups. But for CycE the regulating function depends on Rb, which is marginalized out in our feature set, and also itself depends on an extracellular signal. Hence, the conditional probability constraints for CycE are known only for the setup of the features that determine the output of the Boolean regulating function independent of the other regulator values.

In our comparison analysis, $a^{k_{i}}_{i}(k_{1},\allowbreak \dots, k_{i-1},\allowbreak k_{i+1}, \allowbreak \dots,\allowbreak k_{m})$ for each gene/protein in Eq. (15) is set to one for the feature value setups that determine the Boolean regulating output regardless of the context. But since the observed data are not fully described by these functions, and the system has uncertainty, we let the possible range for the slackness variables in Eq. (15) be [0,1).

We now continue the examples on two of the mammalian cell-cycle network nodes, CycB and CycE. For CycB the following constraints on the prior distribution are extracted from its regulating function:

$$\begin{aligned} & E_{\boldsymbol{p}}[P(v_{10}=0|v_{8}=1)]\geq 1-\epsilon_{1} \\ & E_{\boldsymbol{p}}[P(v_{10}=0|v_{7}=1)]\geq 1-\epsilon_{2} \\ & E_{\boldsymbol{p}}[P(v_{10}=1|v_{7}=0,v_{8}=0)]\geq 1-\epsilon_{3}. \end{aligned} $$

For CycE, one of its regulators is Rb (*v*_2_), which is not included in the feature set, i.e. not observed, but is known to be down-regulated in the mutated (cancerous) case. Thus, the set of constraints extracted from the regulating function of CycE for the normal case includes only

$$\begin{aligned} & E_{\boldsymbol{p}}[P(v_{4}=0|v_{3}=0)]\geq 1-\epsilon_{1} \end{aligned} $$

and for the mutated case consists of

$$\begin{aligned} & E_{\boldsymbol{p}}[P(v_{4}=0|v_{3}=0)]\geq 1-\epsilon_{1} \\ & E_{\boldsymbol{p}}[P(v_{4}=1|v_{3}=1)]\geq 1-\epsilon_{2}. \end{aligned} $$

As another example, for the TP53 network, the set of constraints extracted from the regulating functions in Table 2 for the normal case are shown in the left panel of Table 3.

**Table 3:** The set of constraints extracted from the regulating functions and pathways for the TP53 network. Constraints extracted from the Boolean regulating functions in Table 2 corresponding to the pathway in Fig. 3(b) used in MKDIP-E, MKDIP-D, MKDIP-R (left). Constraints extracted based on [36] from the pathway in Fig. 3(b) used in RMEP, RMDIP, REMLP (right)

(a) MKDIP Constraints		(b) Constraints in Methods of [36]	
Node	Constraint	Node	Constraint
*v*_2_	*E*_***p***_[*P*(*v*_2_=0|*v*_4_=1)]≥1−*ε*_1_	*v*_2_	*E*_***p***_[*P*(*v*_2_=0|*v*_4_=1)]≥1−*ε*_1_
*v*_2_	*E*_***p***_[*P*(*v*_2_=1|*v*_4_=0)]≥1−*ε*_2_	*v*_5_	*E*_***p***_[*P*(*v*_5_=1|*v*_2_=0,*v*_4_=1)]≥1−*ε*_2_
*v*_5_	*E*_***p***_[*P*(*v*_5_=0|*v*_2_=1)]≥1−*ε*_3_		
*v*_5_	*E*_***p***_[*P*(*v*_5_=1|*v*_2_=0,*v*_4_=1)]≥1−*ε*_4_		

The first and second constraints for MKDIP in the left panel of Table 3 come from the regulating function of *v*_2_ in Table 2. Although *v*_1_ is an extracellular signal, the value of *v*_4_ imposes two constraints on the value of *v*_2_. But the regulating function of *v*_4_ in Table 2 only depends on *v*_3_, which is not included in our feature set, so we have no imposed constraints on the conditional probability from its regulating function. The other two constraints for MKDIP in the left panel of Table 3 are extracted from the regulating function of *v*_5_ in Table 2. Although *v*_3_ is not included in the observed features, for two setups of its regulators, (*v*_2_=1) and (*v*_2_=0,*v*_4_=1), the value of *v*_5_ can be determined, so the constraint is imposed on the prior distribution from the regulating function. For comparison, the constraints extracted from the pathway in Fig. 3(b) based on the method of [36] are provided in the right panel of Table 3.

### Performance comparison in classification setup

For both the mammalian cell cycle and TP53 problems, the performance of 11 methods are compared for classification performance. OBC with the Jeffreys’ prior, OBC with our previous prior construction methods in [36] (RMEP, RMDIP, REMLP), OBC with our proposed general framework of constraints (MKDIP-E, MKDIP-D, MKDIP-R), and also well known methods including Histogram rule (Hist), CART [55], Random Forest (RF)[56], and Support Vector Machine classification (SVM) [57, 58]. Also, for all the Bayesian methods using OBC, the posterior mean of the parameters’ distance from the true parameters is calculated and compared. The samples from the true distributions are stratified fixing two different class prior probabilities. Following [36], we assume that $\max _{i}p_{i}^{y,true},~{for} ~y\in \{0,1\}$, is known within a +/−5*%* interval (can come from existing population statistics in practice). Two simulation scenarios are performed: one assuming the complete knowledge of the optimal precision factors [36] $\alpha _{0}^{y}=\sum _{i=1}^{b}\alpha _{i}^{y}, y\in \{0,1\}$ for prior construction methods (oracle precision factor); and the other estimating the optimal precision factor from the observed data itself. Two class prior probabilities, *c*=0.6 and *c*=0.5, are considered. Along with the true class-conditional SSDs of the two classes, the corresponding Bayes error corresponds to the best performance that any classification rule for that classification problem (feature-label distribution) can yield. Fixing *c* and the true class-conditional bin probabilities, *n* sample points by stratified sampling (*n*_0_=⌈*c* *n*⌉ sample points from class 0 and *n*_1_=*n*−*n*_0_ sample points from class 1) are taken for prior construction (if used by the method), classifier training, and posterior distribution calculations. Then the designed classifier’s true classification error is calculated for all classification methods. The posterior mean of parameter distance from the true parameter (true steady-state bin probabilities vector) is calculated based on $\sum _{y=0}^{1}||\boldsymbol {\alpha }^{y\ast }/\alpha _{0}^{y\ast }-\boldsymbol {p}^{y}||^{2}$, where ***α*** ^*y*∗^ and ***p*** ^*y*^ represent the parameters of the posterior distribution and true bin probabilities vector for class *y*, respectively. For each fixed *c* and *n*, 800 Monte Carlo repetitions are done to calculate the expected classification errors and posterior distances from the true parameters for each parameter setup. For REMLP and MKDIP-R, which use a fraction of data in their prior construction procedure, 10 data points from each class are used for prior construction, and all for the inference and posterior calculation (here the number of data points used for prior construction is not fine-tuned, but a small number is chosen to avoid overfitting). The overall procedure taken for a fixed classification problem and a fixed sample size (fixed *n*) in each Monte Carlo repetition is as follows:

- The true bin probabilities ***p*** ^0^ and ***p*** ^1^ are fixed.
- *n*_0_ and *n*_1_ are determined using *c* as *n*_0_=⌈*c* *n*⌉ and *n*−*n*_0_.
- Observations (training data) are randomly sampled from the multinomial distribution for each class, i.e. $(U^{y}_{1},\ldots,U^{y}_{b})\sim \mathcal {M}ult(\boldsymbol {p}^{y};n_{y})$, for *y*∈{0,1}.
- 10 data points are randomly taken from the training data points of each class to be used in the prior construction methods that utilize partial data (REMLP and MKDIP-R)
- All the classification rules are trained based on their constructed prior (if applicable to that classification rule) and the training data.
- The classification errors associated with the classifiers are computed using ***p*** ^0^ and ***p*** ^1^. Also for the Bayesian methods, the posterior probability mass (mean) distance from the true parameters (true bin probabilities, ***p*** ^0^ and ***p*** ^1^) is calculated.

The regularization parameter *λ*_1_ is set to 0.5, and *λ*_2_ is set to 0.25 and 0.5 for the mammalian cell cycle classification problem and the TP53 classification problem, respectively. The results of expected classification error and posterior mean distance from the true parameters for the mammalian cell-cycle network are shown in Tables 4 and 6, respectively. Tables 5 and 7 contain the results of expected classification error and posterior mean distance from the true parameters for the TP53 network.

**Table 4:** Expected true error of different classification rules for the mammalian cell-cycle network. The constructed priors are considered using two precision factors: optimal precision factor (left) and estimated precision factor (right), with *c*=0.5, and *c*=0.6, where the minimum achievable error (Bayes error) is denoted by *E* *r* *r*_*Bayes*_

(a) *c*=0.5, optimal precision factor, *E* *r* *r*_*Bayes*_=0.2648						(b) *c*=0.5, estimated precision factor, *E* *r* *r*_*Bayes*_=0.2648					
Method/ *n*	30	60	90	120	150	Method/ *n*	30	60	90	120	150
Hist	0.3710	0.3423	0.3255	0.3155	0.3081	Hist	0.3710	0.3423	0.3255	0.3155	0.3081
CART	0.3326	0.3195	0.3057	0.3031	0.2975	CART	0.3326	0.3195	0.3057	0.3031	0.2975
RF	0.3359	0.3160	0.3015	0.2991	0.2933	RF	0.3359	0.3160	0.3015	0.2991	0.2933
SVM	0.3359	0.3112	**0.2977**	0.2959	0.2940	SVM	0.3359	0.3112	0.2977	0.2959	0.2940
Jeffreys’	0.3710	0.3423	0.3255	0.3155	0.3081	Jeffreys’	0.3710	0.3423	0.3255	0.3155	0.3081
RMEP	0.3236	0.3070	0.3010	0.2946	0.2910	RMEP	0.3315	0.3059	0.2985	0.2963	0.2930
RMDIP	0.3236	0.3070	0.3010	0.2946	0.2910	RMDIP	0.3314	0.3060	0.2986	0.2965	0.2931
REMLP	0.3425	0.3264	0.3146	0.3067	0.3011	REMLP	0.3488	0.3352	0.3202	0.3101	0.3048
MKDIP-E	0.3221	0.3070	0.3010	0.2949	0.2910	MKDIP-E	0.3313	0.3056	0.2982	0.2962	0.2929
MKDIP-D	0.3232	0.3070	0.3010	0.2952	0.2910	MKDIP-D	0.3315	0.3061	0.2986	0.2965	0.2931
MKDIP-R	**0.3149**	**0.3028**	0.2985	**0.2943**	**0.2907**	MKDIP-R	**0.3205**	**0.3041**	**0.2969**	**0.2947**	**0.2919**
(c) *c*=0.6, optimal precision factor, *E* *r* *r*_*Bayes*_=0.31						(d) *c*=0.6, estimated precision factor, *E* *r* *r*_*Bayes*_=0.31					
Method/ *n*	30	60	90	120	150	Method/ *n*	30	60	90	120	150
Hist	0.3622	0.3608	0.3624	0.3641	0.3652	Hist	0.3622	0.3608	0.3624	0.3641	0.3652
CART	0.3554	0.3556	0.3507	0.3510	0.3447	CART	0.3554	0.3556	0.3507	0.3510	0.3447
RF	0.3524	0.3514	0.3467	0.3476	0.3420	RF	0.3524	0.3514	0.3467	0.3476	0.3420
SVM	0.3735	0.3684	0.3615	0.3602	0.3544	SVM	0.3735	0.3684	0.3615	0.3602	0.3544
Jeffreys’	0.3620	0.3559	0.3519	0.3502	0.3472	Jeffreys’	0.3620	0.3559	0.3519	0.3502	0.3472
RMEP	**0.3415**	0.3385	**0.3394**	**0.3390**	**0.3386**	RMEP	0.3528	0.3415	0.3407	0.3388	0.3378
RMDIP	**0.3415**	**0.3383**	**0.3394**	**0.3390**	**0.3386**	RMDIP	0.3529	0.3415	0.3408	0.3388	0.3378
REMLP	0.3666	0.3625	0.3587	0.3558	0.3530	REMLP	0.3700	0.3650	0.3603	0.3578	0.3546
MKDIP-E	**0.3415**	0.3384	**0.3394**	**0.3390**	**0.3386**	MKDIP-E	0.3525	**0.3413**	**0.3405**	**0.3387**	**0.3377**
MKDIP-D	**0.3415**	0.3386	**0.3394**	**0.3390**	**0.3386**	MKDIP-D	0.3532	0.3418	0.3409	0.3389	0.3379
MKDIP-R	0.3437	0.3409	0.3404	0.3401	0.3389	MKDIP-R	**0.3486**	0.3416	0.3416	0.3402	0.3387

The lowest error for each sample size is written in bold

**Table 5:** Expected true error of different classification rules for the TP53 network. The constructed priors are considered using two precision factors: optimal precision factor (left) and estimated precision factor (right), with *c*=0.5, and *c*=0.6, where the minimum achievable error (Bayes error) is denoted by *E* *r* *r*_*Bayes*_

(a) *c*=0.5, optimal precision factor, *E* *r* *r*_*Bayes*_=0.3146						(b) *c*=0.5, estimated precision factor, *E* *r* *r*_*Bayes*_=0.3146					
Method/ *n*	15	30	45	60	75	Method/ *n*	15	30	45	60	75
Hist	0.3586	0.3439	0.3337	0.3321	0.3296	Hist	0.3586	0.3439	**0.3337**	0.3321	0.3296
CART	0.3633	0.3492	0.3350	0.3314	0.3295	CART	0.3633	0.3492	0.3350	**0.3314**	0.3295
RF	0.3791	0.3574	0.3461	0.3400	0.3362	RF	0.3791	0.3574	0.3461	0.3400	0.3362
SVM	0.3902	0.3481	0.3433	0.3324	0.3322	SVM	0.3902	0.3481	0.3433	0.3324	0.3322
Jeffreys’	0.3809	0.3439	0.3457	0.3321	0.3334	Jeffreys’	0.3809	0.3439	0.3457	0.3321	0.3334
RMEP	0.3399	0.3392	0.3360	0.3315	0.3328	RMEP	0.3791	0.3489	0.3377	0.3329	0.3302
RMDIP	0.3399	0.3392	0.3360	0.3315	0.3328	RMDIP	0.3789	0.3490	0.3378	0.3329	0.3302
REMLP	0.3405	**0.3340**	**0.3320**	**0.3292**	0.3287	REMLP	**0.3417**	**0.3372**	0.3350	0.3318	0.3292
MKDIP-E	**0.3397**	0.3398	0.3351	0.3306	0.3297	MKDIP-E	0.3675	0.3470	0.3373	0.3326	0.3298
MKDIP-D	**0.3397**	0.3398	0.3347	0.3306	0.3297	MKDIP-D	0.3668	0.3472	0.3374	0.3327	0.3298
MKDIP-R	0.3435	0.3354	0.3321	0.3295	**0.3283**	MKDIP-R	0.3471	0.3402	0.3349	0.3316	**0.3287**
(c) *c*=0.6, optimal precision factor, *E* *r* *r*_*Bayes*_=0.2691						(d) *c*=0.6, estimated precision factor, *E* *r* *r*_*Bayes*_=0.2691					
Method/ *n*	15	30	45	60	75	Method/ *n*	15	30	45	60	75
Hist	0.3081	0.2965	0.2906	0.2883	0.2846	Hist	0.3081	0.2965	0.2906	0.2883	0.2846
CART	0.3173	0.2988	0.2882	0.2846	**0.2796**	CART	0.3173	0.2988	0.2882	0.2846	**0.2796**
RF	0.3333	0.3035	0.2946	0.2850	0.2842	RF	0.3333	0.3035	0.2946	0.2850	0.2842
SVM	0.3322	0.3091	0.2991	0.2926	0.2857	SVM	0.3322	0.3091	0.2991	0.2926	0.2857
Jeffreys’	0.3105	0.2936	0.2860	**0.2828**	0.2819	Jeffreys’	0.3105	0.2936	**0.2860**	**0.2828**	0.2819
RMEP	**0.2924**	0.2922	0.2847	0.2843	0.2835	RMEP	0.3346	0.3024	0.2894	0.2860	0.2823
RMDIP	**0.2924**	0.2922	0.2847	0.2843	0.2835	RMDIP	0.3344	0.3023	0.2895	0.2858	0.2823
REMLP	0.3003	**0.2908**	0.2869	0.2839	0.2832	REMLP	**0.3054**	**0.2930**	0.2910	0.2870	0.2850
MKDIP-E	**0.2924**	0.2909	**0.2837**	0.2851	0.2837	MKDIP-E	0.3341	0.3025	0.2898	0.2864	0.2822
MKDIP-D	**0.2924**	0.2909	**0.2837**	0.2851	0.2837	MKDIP-D	0.3347	0.3024	0.2898	0.2862	0.2822
MKDIP-R	0.3032	0.2917	0.2868	0.2843	0.2825	MKDIP-R	0.3096	0.2981	0.2910	0.2869	0.2849

The lowest error for each sample size is written in bold

The best performance (with the lowest error in Tables 4 and 5, and lowest distance in Tables 6 and 7) for each sample size, are written in bold. For the mammalian cell-cycle network, MKDIP methods show the best (or as good as the best) performance in all the scenarios in terms of both the expected classification error and posterior parameter estimates. For the TP53 network, MKDIP methods show the best performances in posterior parameter estimates, and are competitive with the previous knowledge-driven prior construction methods in terms of the expected classification error.

**Table 6:** Expected difference between the true model (for mammalian cell-cycle network) and estimated posterior probability masses. Optimal precision factor (left) and estimated precision factor (right), with *c*=0.5, and *c*=0.6

(a) *c*=0.5, optimal precision factor						(b) *c*=0.5, estimated precision factor					
Method/ *n*	30	60	90	120	150	Method/ *n*	30	60	90	120	150
Jeffreys’	0.2155	0.1578	0.1300	0.1134	0.1010	Jeffreys’	0.2155	0.1578	0.1300	0.1134	0.1010
RMEP	0.1591	0.1293	0.1126	0.1020	0.0912	RMEP	0.1761	**0.1381**	**0.1177**	0.1032	**0.0943**
RMDIP	0.1591	0.1294	0.1126	0.1020	0.0912	RMDIP	0.1761	**0.1381**	**0.1177**	0.1032	**0.0943**
REMLP	0.1863	0.1436	0.1225	0.1088	0.0970	REMLP	0.2060	0.1607	0.1315	0.1120	0.1019
MKDIP-E	0.1589	0.1293	0.1126	0.1019	0.0911	MKDIP-E	0.1760	**0.1381**	**0.1177**	**0.1031**	**0.0943**
MKDIP-D	0.1591	0.1293	0.1126	0.1020	0.0912	MKDIP-D	0.1761	**0.1381**	**0.1177**	0.1032	**0.0943**
MKDIP-R	**0.1563**	**0.1283**	**0.1118**	**0.1012**	**0.0907**	MKDIP-R	**0.1742**	0.1392	0.1184	0.1036	0.0949
(c) *c*=0.6, optimal precision factor						(d) *c*=0.6, estimated precision factor					
Method/ *n*	30	60	90	120	150	Method/ *n*	30	60	90	120	150
Jeffreys’	0.2183	0.1595	0.1322	0.1146	0.1027	Jeffreys’	0.2183	0.1595	0.1322	0.1146	0.1027
RMEP	0.1628	0.1332	0.1154	0.1039	0.0946	RMEP	0.1805	**0.1408**	0.1201	**0.1061**	**0.0961**
RMDIP	0.1628	0.1333	0.1154	0.1039	0.0947	RMDIP	0.1805	**0.1408**	0.1201	**0.1061**	**0.0961**
REMLP	0.1867	0.1471	0.1247	0.1101	0.0990	REMLP	0.2065	0.1635	0.1346	0.1166	0.1036
MKDIP-E	0.1627	0.1332	0.1154	0.1038	0.0946	MKDIP-E	**0.1804**	**0.1408**	**0.1200**	**0.1061**	**0.0961**
MKDIP-D	0.1628	0.1332	0.1154	0.1039	0.0946	MKDIP-D	0.1805	**0.1408**	0.1201	**0.1061**	**0.0961**
MKDIP-R	**0.1598**	**0.1317**	**0.1144**	**0.1032**	**0.0940**	MKDIP-R	0.1814	0.1421	0.1207	0.1065	0.0965

The lowest distance for each sample size is written in bold

**Table 7:** Expected difference between the true model (for TP53 network) and estimated posterior probability masses. Optimal precision factor (left) and estimated precision factor (right), with *c*=0.5, and *c*=0.6

(a) *c*=0.5, optimal precision factor						(b) *c*=0.5, estimated precision factor					
Method/ *n*	15	30	45	60	75	Method/ *n*	15	30	45	60	75
Jeffreys’	0.2285	0.1716	0.1429	0.1242	0.1114	Jeffreys’	0.2285	0.1716	0.1429	0.1242	0.1114
RMEP	0.1427	0.1165	0.1051	0.0934	0.0880	RMEP	0.2218	0.1578	**0.1280**	0.1095	**0.0981**
RMDIP	0.1424	0.1163	0.1048	0.0932	**0.0878**	RMDIP	0.2217	0.1575	0.1281	**0.1094**	**0.0981**
REMLP	0.1698	0.1337	0.1199	0.1091	0.0985	REMLP	0.1845	0.1505	0.1366	0.1235	0.1133
MKDIP-E	0.1412	0.1161	0.1050	0.0933	0.0880	MKDIP-E	0.2149	0.1565	0.1282	0.1096	**0.0981**
MKDIP-D	**0.1407**	**0.1158**	**0.1047**	**0.0931**	**0.0878**	MKDIP-D	0.2149	0.1564	0.1281	0.1096	**0.0981**
MKDIP-R	0.1564	0.1247	0.1118	0.1031	0.0930	MKDIP-R	**0.1733**	**0.1410**	0.1281	0.1171	0.1082
(c) *c*=0.6, optimal precision factor						(d) *c*=0.6, estimated precision factor					
Method/ *n*	15	30	45	60	75	Method/ *n*	15	30	45	60	75
Jeffreys’	0.2319	0.1723	0.1438	0.1262	0.1137	Jeffreys’	0.2319	0.1723	0.1438	0.1262	0.1137
RMEP	0.1476	0.1222	0.1090	0.0987	0.0923	RMEP	0.2182	0.1599	0.1304	**0.1144**	0.1032
RMDIP	0.1474	0.1220	0.1087	0.0985	0.0921	RMDIP	0.2179	0.1597	0.1303	**0.1144**	**0.1031**
REMLP	0.1751	0.1332	0.1192	0.1077	0.0980	REMLP	0.1937	0.1522	0.1363	0.1235	0.1144
MKDIP-E	0.1457	0.1215	0.1086	0.0985	0.0922	MKDIP-E	0.2165	0.1586	0.1304	0.1147	0.1036
MKDIP-D	**0.1452**	**0.1211**	**0.1084**	**0.0983**	**0.0920**	MKDIP-D	0.2164	0.1585	0.1303	0.1147	0.1035
MKDIP-R	0.1574	0.1217	0.1093	0.1010	0.0926	MKDIP-R	**0.1758**	**0.1418**	**0.1274**	0.1158	0.1086

The lowest distance for each sample size is written in bold

### Performance comparison in mixture setup

The performance of the OBC with different prior construction methods, including OBC with the Jeffreys’ prior, OBC with prior constructions methods of [36] (RMEP, RMDIP, REMLP), and OBC with the general framework of constraints (MKDIP-E, MKDIP-D, MKDIP-R), are further compared in the mixture setup with missing labels, for both the mammalian cell-cycle and the TP53 systems. Also, the OBC with prior distribution centered on the true parameters with a relatively low variance (hereinafter abbreviated as PDCOTP method in Tables 8 and 9) is considered as the comparison baseline, though it is not a practical method. Similar to the classification problems, we assume that only two components (classes) exist, normal and mutated (cancerous). Here, *c*_0_ is fixed at 0.6 (*c*_1_=1−*c*_0_=0.4), but the sampling is not stratified. The component-conditional SSDs (bin probabilities) for the two components are as before in the classification problem, i.e. the same as the class-conditional SSDs in the classification problem.

**Table 8:** Expected errors of different Bayesian classification rules in the mixture model for the mammalian cell-cycle network. Expected true error (left) and expected error on unlabeled training data (right), with *c*_0_=0.6

Method/ *n*	30	60	90	120	150	Method/ *n*	30	60	90	120	150
PDCOTP	**0.3216**	**0.3246**	**0.3280**	0.3309	0.3334	PDCOTP	**0.3236**	**0.3270**	**0.3314**	0.3355	0.3339
Jeffreys’	0.4709	0.4743	0.4704	0.4675	0.4654	Jeffreys’	0.4751	0.4621	0.4681	0.4700	0.4645
RMEP	0.3417	0.3340	0.3307	0.3300	0.3299	RMEP	0.3447	0.3409	0.3366	0.3323	0.3316
RMDIP	0.3408	0.3336	0.3300	0.3305	0.3301	RMDIP	**0.3442**	0.3404	0.3342	0.3344	0.3343
REMLP	0.3754	0.3835	0.3882	0.3857	0.3844	REMLP	0.3748	0.3821	0.3908	0.3826	0.3812
MKDIP-E	0.3411	0.3341	**0.3297**	0.3297	0.3306	MKDIP-E	0.3457	0.3386	0.3351	0.3312	0.3320
MKDIP-D	**0.3407**	**0.3330**	0.3306	0.3304	0.3303	MKDIP-D	0.3482	0.3387	0.3381	0.3342	0.3334
MKDIP-R	0.3457	0.3342	0.3299	**0.3286**	**0.3289**	MKDIP-R	0.3449	**0.3343**	**0.3330**	**0.3306**	**0.3275**

The lowest error for each sample size and the lowest error among practical methods is written in bold

**Table 9:** Expected errors of different Bayesian classification rules in the mixture model for the TP53 network. Expected true error (left) and expected error on unlabeled training data (right), with *c*_0_=0.6

Method/ *n*	15	30	45	60	75	Method/ *n*	15	30	45	60	75
PDCOTP	**0.2746**	**0.2824**	**0.2829**	**0.2996**	**0.2960**	PDCOTP	**0.2762**	**0.2818**	**0.2900**	**0.3027**	**0.2900**
Jeffreys’	0.4204	0.4324	0.4335	0.4432	0.4361	Jeffreys’	0.4220	0.4314	0.4381	0.4419	0.4348
RMEP	**0.3274**	**0.3204**	0.3327	**0.3402**	0.3422	RMEP	**0.3471**	0.3350	0.3487	0.3543	0.3529
RMDIP	0.3297	0.3260	0.3327	0.3406	0.3432	RMDIP	0.3504	0.3423	0.3496	0.3551	0.3545
REMLP	0.3637	0.3687	0.3706	0.3658	0.3653	REMLP	0.3489	0.3579	0.3709	0.3593	0.3556
MKDIP-E	0.3312	0.3246	0.3322	0.3428	0.3386	MKDIP-E	0.3502	0.3378	0.3486	0.3585	0.3492
MKDIP-D	0.3321	**0.3204**	**0.3306**	0.3436	**0.3366**	MKDIP-D	0.3551	**0.3329**	**0.3473**	0.3570	0.3475
MKDIP-R	0.3872	0.3749	0.3667	0.3607	0.3586	MKDIP-R	0.3613	0.3583	0.3589	**0.3539**	**0.3462**

The lowest error for each sample size and the lowest error among practical methods is written in bold

For each sample point, first the label (*y*) is generated from a Bernoulli distribution with success probability *c*_1_, and then the bin observation is generated given the label, from the corresponding class-conditional SSD (class conditional bin probabilities vector, ***p*** ^*y*^), i.e. the bin observation is a sample from a categorical distribution with parameter vector ***p*** ^*y*^ but the label is hidden for the inference chain and classifier training. *n* sample points are generated and fed into the Gibbs inference chain with different priors from the different prior construction methods. Then the OBC is calculated based on Eq. 9. For each sample size, 400 Monte Carlo repetitions are done to calculate the expected true error and the error of classifying the unlabeled observed data used for the inference itself.

To have a fair comparison of different methods’ class-conditional prior probability construction, we assume that we have a rough idea of the mixture weights (class probabilities). In practice this can come from existing population statistics. That is, the Dirichlet prior distribution over the mixture weights (class probabilities) parameters, ***ϕ*** in $\mathcal {D}(\boldsymbol {\phi })$, are sampled in each iteration from a uniform distribution that is centered on the true mixture weights vector +/−10*%* interval, and fixed for all the methods in that repetition. For the REMLP and MKDIP-R that need labeled data in their prior construction procedure, the predicted labels from using the Jeffreys’ prior are used and one fourth of the data points are used in prior construction for these two methods, and all for inference. The reason for using a larger number of data points in prior construction within the mixture setup compared to the classification setup is that in the mixture setup, data points are missing their true class labels, and the initial label estimates may be inaccurate. One can use a relatively larger number of data points in prior construction, which still avoids overfitting. The regularization parameters *λ*_1_ and *λ*_2_ are set as in the classification problem. Optimal precision factors are used for all prior construction methods. The results are shown in Tables 8 and 9 for the mammalian cell-cycle and TP53 models, respectively. The best performance (lowest error) for each sample size and the best performance among practical methods (all other than PDCOTP), if different, is written in bold. As can be seen from the tables, in most cases the MKDIP methods have the best performance among the practical methods. With larger sample sizes, MKDIP-R even outperforms PDCOTP in the mammalian cell-cycle system.

### Performance comparison on a real data set

**Fig. 4:**
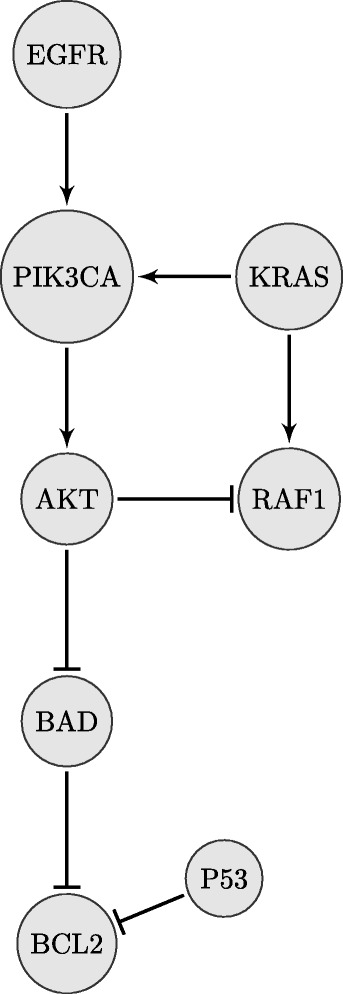
Signaling pathways corresponding to NSCLC classification. The pathways are collected from KEGG Pathways for NSCLC and PI3K-AKT pathways, and from [63]

In this section the performance of the proposed methods are examined on a publicly available gene expression dataset. Here, we have considered the classification of two subtypes of non-small cell lung cancer (NSCLC), lung adenocarcinoma (LUA) versus lung squamous cell carcinoma (LUS). Lung cancer is the second most commonly diagnosed cancer and the leading cause of cancer death in both men and women in the United States [59]. About 84% of lung cancers are NSCLC [59] and LUA and LUS combined account for about 70% of lung cancers based on the American Cancer Society statistics for NSCLC. We have downloaded LUA and LUS datasets (both labeled as TCGA provisional) in the form of mRNA expression *z*-scores (based on RNA-Seq profiling) from the public database cBioPortal [60, 61] for the patient sets tagged as “All Complete Tumors", denoting the set of all tumor samples that have mRNA and sequencing data. The two datasets for LUA and LUS consist of 230 and 177 sample points, respectively. We have quantized the data into binary levels based on the following preprocessing steps. First, to remove the bias for each patient, each patient’s data are normalized by the mean of the *z*-scores of a randomly selected subset from the list of the recurrently mutated genes (half the size of the list) from the MutSig [62] (directly provided by cBioPortal). Then, a two component Gaussian mixture model is fit to each gene in each data set, and the normalized data are quantized by being assigned to one component, namely 0 or 1 (1 being the component with higher mean). We confine the feature set to {EGFR,PIK3CA,AKT,KRAS,RAF1,BAD,P53,BCL2} which are among the genes in the most relevant signaling pathways to the NSCLC [63]. These genes are altered, in different forms, in 86% and 89% of the sequenced LUA and LUS tumor samples on the cBioPortal, respectively. There are 256 bins in this classification setting, since the feature set consists of 8 genes. The pathways relevant to the NSCLC classification problem considered here are collected from KEGG [64, 65] Pathways for NSCLC and PI3K-AKT signaling pathways, and also from [63], as shown in Fig. 4. The corresponding regulating functions are shown in Table 10.

**Table 10:** Regulating functions corresponding to the signaling pathways in Fig. 4. In the Boolean functions {AND, OR, NOT}={∧,∨,−}

Gene	Node name	Boolean regulating function
EGFR	*v*_1_	-
PIK3CA	*v*_2_	*v*_1_∨*v*_4_
AKT	*v*_3_	*v*_2_
KRAS	*v*_4_	-
RAF1	*v*_5_	$v_{4} \wedge \overline {v_{3}}$
BAD	*v*_6_	$\overline {v_{3}}$
P53	*v*_7_	-
BCL2	*v*_8_	$\overline {v_{6}} \vee \overline {v_{7}}$

The informative prior construction methods utilize the knowledge in the pathways in Fig. 4, and the MKDIP methods also use the regulating relationships in Table 10 in order to construct prior distributions. The incidence rate of the two subtypes, LUA and LUS, varies based on demographic factors. Here, we approximate the class probability *c*=*P*(*Y*=LUA) as *c*≈0.57, based on the latest statistics of the American Cancer Society for NSCLC, and also based on a weighted average of the rates for 11 countries given in [66]. In each Monte Carlo repetition, *n* sample points by stratified sampling, i.e. *n*_0_=⌈*c* *n*⌉ and *n*_1_=*n*−*n*_0_ sample points, are randomly taken from preprocessed LUA (class 0) and LUS (class 1) datasets, respectively, for prior construction (if used by the method) and classifier training, and the rest of the sample points are held out for error estimation. For each *n*, 400 Monte Carlo repetitions are done to calculate the expected classification error. In the prior construction methods, first the optimization is solved for both classes with the precision factors $\alpha ^{y}_{0}=200, y\in \{0,1\}$, and then their optimal values are estimated using the training points. For REMLP and MKDIP-R, which use a fraction of the training data in their prior construction procedure, min(20,max(6,⌊0.25*n*_*y*_⌋)) sample points from the training data of each class (*y*∈{0,1}) are used for prior construction, and all the training data are used for inference. The regularization parameters *λ*_1_ and *λ*_2_ are set to 0.5 and 0.25, respectively. The results are shown in Table 11. In the table, the best performance among Hist, CART, RF and SVM is shown as Best Non Bayesian method. Best RM represents the best performance among RMEP, RMDIP, and REMLP. Best MKDIP denotes the best performance among the MKDIP methods.

**Table 11:** Expected error of different classification rules calculated on a real dataset. The classification is between LUA (class 0) and LUS (class 1), with *c*=0.57

Method/ *n*	34	74	114	134	174
Best Non Bayesian	0.1764	0.1574	0.1473	0.1426	0.1371
Jeffreys’	0.1766	0.1574	0.1476	0.1425	0.1371
Best RM	0.1426	0.1289	0.1164	0.1083	0.1000
Best MKDIP	**0.1401**	**0.1273**	**0.1162**	**0.1075**	**0.0998**

The best performing rule for each sample size is written in bold. As can be seen from the table, OBC with MKDIP prior construction methods has the best performance among the classification rules. It is also clear that the classification performance can be significantly improved when pathway prior knowledge is integrated for constructing prior probabilities, especially when the sample size is small.

### Implementation remarks

The results presented in this paper are based on Monte Carlo simulations, where thousands of optimization problems are solved for each sample size for each problem. Thus, the regularization parameters and the number of sample points used in prior construction are preselected for each problem. One can use cross validation to set these parameters in a specific application. It has been shown in [36] that by assuming precision factors greater than 1 ($\alpha _{0}^{y}>1, y\in \{0,1\}$), all three objective functions used are convex for the class of Dirichlet prior probabilities for multinomial likelihood functions. But unfortunately, we cannot guarantee the convexity of the feasible space due to the convolved constraints. Therefore, we have employed algorithms for nonconvex optimization problems and there is no guarantee of convergence to the global optimum. The method used for solving the optimization framework of the prior construction is based on the interior-point algorithm for nonlinear constrained optimization [67, 68] implemented in the fmincon function in MATLAB. In this paper, since the interest is in classification problems with small training sample sizes (which is often the case in bioinformatics) and also due to Monte Carlo simulations, we have only shown performance results on small networks with only a few genes. In practice, there would be no problem using the proposed method for larger networks, since there would then be a single one-time analysis. One should also note that with small sample sizes, one needs feature selection to keep the number of features small. In the experiments in this paper, feature selection is automatically done by focusing on the most relevant network by biological prior knowledge.

## Conclusion

Bayesian methods have shown promising performance in classification problems in the presence of uncertainty and small sample sizes, which often occur in translational genomics problems. The impediment in using these methods is prior construction to integrate existing prior biological knowledge. In this paper we have proposed a knowledge-driven prior construction method with a general framework of mapping prior biological knowledge into a set of constraints. Knowledge can come from biological signaling pathways and other population studies, and be translated into constraints over conditional probabilities. This general scheme includes the previous approaches of using biological prior knowledge in prior construction. Here, the superior performance of this general scheme is shown on two important pathway families, the mammalian cell-cycle pathway and the pathway centering around TP53. In addition, prior construction and the OBC are extended to a mixture model, where data sets are with missing labels. Moreover, comparisons on a publicly available gene expression dataset show that classification performance can be significantly improved for small sample sizes when corresponding pathway prior knowledge is integrated for constructing prior probabilities.
